# Territorial Compensation for Injuries

**Published:** 1912-04-06

**Authors:** 


### THE ROYAL ARMY MEDICAL CORPS SECTION.

### Territorial Compensation for Injuries.

When performing military duty, especially during the camping season, both officers and men of the Territorial Force are liable to meet with injuries and be incapacitated, either permanently or temporarily, from following their employment. Some of our insurance companies have naturally taken steps to provide for this contingency by offering policies to cover all accidents which may arise in the course of a Territorial's military work, the premiums ranging from 2s. 6d. to 10s. per annum for foot soldiers, and from 5s. to 20s. per annum for artillery and mounted men. These rates are in every way moderate, and the prudent Territorial might do worse than avail himself of them, but we are glad to see that the military authorities have recognised their responsibilities in this matter and have allowed compensation to officers and men for injuries received and illness contracted during any period of military duty. The amount of this compensation and the conditions governing it are laid down in the Territorial Forces Regulations. Attention has been drawn to a certain dubiety about the paragraphs dealing with the subject, and it has been urged that the Government should abandon them and accept all responsibility under the Employers' Liability Act during camp training. We are not sure that this is good advice, as the terms of compensation offered to officers and men seem to us reasonable and fair.

### How the Territorial is Treated.

Speaking generally, the basis on which the Territorial officer and soldier is treated is that laid down by Lord Lucas in a statement made last year to the House of Lords, when he said that the War Office deals with the Territorials in exactly the same way as if they were Regulars. Taking first the case of permanent incapacity from injury, every regular soldier discharged from the Army on this account has a claim for a pension; so in the same way, according to paragraph 667 of the Territorial Forces Regulations, it is equally clear that all members of the Territorial Force are eligible to receive these disability pensions. The method of procedure for obtaining such a pension is the same in both cases. Each applicant is examined by a board of medical officers and the case is put forward for an Army pension on Army Form B 179, the board giving its opinion as to the amount of disablement present, this being expressed in the terms quarter, half, three-quarters, and total. The finding of this board is sent to the Chelsea Commissioners, with whom the final decision in the case rests. It is they who reject or accept the application, as well as determining the amount of the pension in the cases where one is granted. In view of the very explicit statement made in paragraph 667 of the Territorial Forces Regulations, we do not share the opinion of those who hold that a pension is not guaranteed to a Territorial officer or soldier if permanently disabled when on military duty. We think that his right to it is quite secure.

### Temporary Injuries.

Coming next to injuries that are not permanent, in the case of officers paragraph 658 of the Territorial Forces Regulations states that they are entitled to treatment in a military hospital, and while there to draw pay of their equivalent rank in the regular service, less a daily charge of 2s. 6d. per diem for hospital maintenance. If, however, an officer elects to be treated at home, he gets the pay of his rank while unable to follow his civil employment and reimbursement of medical expenses, it being understood that these grants are for a period not exceeding six months and are at the discretion of the Army Council. Should an officer be laid aside from work for longer than six months, then his case has to be specially considered and the question of permanent disablement gone into in the manner already detailed.

As regards the non-commissioned officers and men, paragraph 659 of the Territorial Forces Regulations deals with them when out for training in camp, or on manœuvres, during an authorised course of instruction or when specially called up for duty, and authorises their admission to a military hospital with the pay of their rank while so detained, provided the injuries are not received through any fault of their own. This residence in the military hospital with pay may be extended, if necessary, to a period of six months, but paragraph 660 lays it down that if pay is being received there will be a deduction of one shilling per diem for hospital maintenance during that time. Even after six months the non-commissioned officers and men may continue to remain in the military hospital should they still be disabled, and they will do so as free patients, their pay, of course, having ceased at the end of the six months. Should the disablement prove to be permanent, then each case will be considered on its merits as to its eligibility for a pension, in the same manner as has been already outlined in the case of officers.

### Some Concessions.

In the case, however, of non-commissioned officers and men, paragraph 662 of the Territorial Forces Regulations provides special terms for them should they decide not to remain in the military hospital, but to go to their own homes. Under it a sum not exceeding 3s. 6d. a day may be granted for a period not exceeding six months while they are unable to resume their trade or calling. To obtain this gratuity it is necessary that the injury should be at once reported to the General Officer Commanding-in-Chief, who will, if he deems it necessary, after perusal of the medical evidence, direct an officer of the Royal Army Medical Corps to report on the case, unless the injured Territorial is in a civil hospital, when a medical certificate from the hospital authorities as to the nature of the injuries will be accepted. It is also very distinctly stated that this gratuity of 3s. 6d. per day will be limited to the period during which the non-commissioned officer or man was shown to have been unable to follow his occupation, and will not be issuable for the time when the individual is in receipt of pay or subsisted in a military hospital. Another very proper concession is conferred by paragraph 664 of the Territorial Forces Regulations, which lays it down that in cases where, although the Territorial is able to follow his trade or calling, medical attendance is necessary in consequence of injury, his actual medical expenses up to a maximum of 3s. 6d. per diem may be repaid, provided the Director-General, Army Medical Service, is satisfied that the disability was contracted in and through the performance of military duty.

So far the conditions laid down refer to injuries received by the Territorial officer and soldier, but provision has been made also for illness, it being, however, laid down as a fixed rule that, as regards illness, a gratuity is not admissible in cases where a disease, though contracted during a period of military duty, has arisen from the ordinary risks of life, to which military duty carries with it no special liability. Barring this proviso, the gratuity of 3s. 6d. per diem may be paid to the non-commissioned officers and men if laid aside by illness that has been proved to the satisfaction of the Director-General of the Army Medical Service to have been contracted through the performance of military duty, but it is absolutely necessary that the claim must be preferred within twelve months of the termination of the military duty in question.

We have gone very fully into this question of compensation to injured Territorials, as the exact state of matters is not generally understood and anxieties are naturally felt upon this point by the parents of those serving in the force. What has been said should make it clear that the military authorities are dealing fairly and considerately with both the officers and the men. One other point we would emphasise, and that is the need for making claims for compensation through the proper channel and in a complete state. Commanding officers of units and regimental medical officers should pay special attention to this, it being remembered that if the gratuity is claimed on account of an injury the application will be forwarded for the decision of the General Officer Commanding-in-Chief, but if on account of illness it will be sent to the War Office. In both cases it should be accompanied by the necessary documents and reports. It is also important for commanding officers to bear in mind that all cases of men who are about to be dismissed from training in a state unfit to resume work (paragraph 663), or who will require medical attendance after dismissal (paragraph 664), should be reported on before the men are finally dismissed.

